# Research progress on microglial pyroptosis and inflammasomes: a comprehensive analysis

**DOI:** 10.3389/fnagi.2025.1582579

**Published:** 2025-06-09

**Authors:** Xiaofang Wang, Zhenyu Li, Bingxiang Ma, Qianfang Jia

**Affiliations:** ^1^Hebei University of Chinese Medicine, Shijiazhuang, China; ^2^School of Medical Engineering, Xinxiang Medical University, Xinxiang, China; ^3^Children’s Brain Disease Diagnosis, Treatment and Rehabilitation Center of the First Affiliated Hospital of Henan University of Chinese Medicine, Zhengzhou, China; ^4^School of Pediatric Medicine, Henan University of Chinese Medicine, Zhengzhou, China; ^5^Department of Children Rehabilitation, First Affiliated Hospital of Xinxiang Medical University, Xinxiang, China; ^6^Xinxiang Key Laboratory of Autism Disease Mechanism Research, Xinxiang, China; ^7^Xinxiang Autism Integration Education Engineering and Technology Research Center, Xinxiang, China

**Keywords:** microglia, pyroptosis, inflammasome, mechanism, neurodegenerative disease, research progress

## Abstract

**Background:**

Microglial pyroptosis and inflammasome activation play critical roles in neurodegenerative diseases, especially Alzheimer’s disease (AD), Parkinson’s disease (PD), and multiple sclerosis (MS). In recent years, substantial attention has been directed toward elucidating their underlying mechanisms, diagnostic approaches, and prognostic implications. This study aimed to analyze the current research landscape, hotspots, and trends in this field.

**Methods:**

Articles published over the past decade on microglial pyroptosis and inflammasomes were retrieved from the Web of Science Core Collection (WoSCC) database. A comprehensive analysis was conducted, and high-impact articles were examined in depth.

**Results:**

A total of 958 articles were included. Among these, 664 originated from China, which also had the highest H-index (68), followed by 147 articles from the United States, with an H-index of 48 and the highest centrality (0.68). Southern Medical University (China) was the leading institution in terms of articles (47) and achieved the highest H-index (19). Journal of Neuroinflammation published the most articles (59) in this field. High-impact studies predominantly focused on the roles of microglial pyroptosis and inflammasomes in neurodegenerative diseases, neuroinflammation and therapeutic intervention strategies. Keywords such as “depression,” “cell death,” “recovery,” and “pathogenesis” emerged as research hotspots over the past 3 years.

**Conclusion:**

Microglial pyroptosis and inflammasome activation have become research hotspots in neurodegenerative disease, with China and the United States leading in article output and research influence in this field. Southern Medical University (China) is the most influential institution, and the *Journal of Neuroinflammation* is the most prolific journal. Current research hotspots emphasize elucidating the pathological mechanisms of microglial pyroptosis and inflammasome activation in neurodegenerative diseases, especially in AD, PD, and MS, and exploring potential therapeutic strategies such as MCC950, quercetin, MicroRNA-7, and melatonin. Future studies are expected to focus on mechanism elucidation, disease specificity, dynamic regulation, targeted interventions, and clinical translation to enhance treatment outcomes and prognosis for neurological disorders.

## 1 Introduction

Microglial pyroptosis and inflammasome activation are critical research topics in neuroimmunology, involved in multiple pathological processes such as neurodegenerative diseases, neuroinflammation, and neurological injury (Li Y. et al., 2024). Pyroptosis is a form of programmed cell death typically associated with inflammation. Inflammasomes are essential intracellular multi-protein complexes that initiate immune responses and regulate inflammatory reactions. Among them, the nucleotide oligomerization domain-, leucine-rich repeat-, and pyrin domain-containing protein 3 (NLRP3) inflammasome is particularly significant in the pyroptotic process. It facilitates the activation of inflammatory caspases and promotes the cleavage of gasdermin D (GSDMD). Additionally, it triggers the release of inflammatory cytokines such as IL-1β and IL-18, thereby orchestrating the inflammatory response and contributing to cellular damage (Fan et al., 2021; Ravichandran and Heneka, 2021; Han et al., 2023). Consequently, pyroptosis and inflammasomes have become focal points of research on inflammation and cellular damage. In recent years, the role of microglial pyroptosis and inflammasomes in neurodegenerative diseases has attracted significant academic attention. Researchers worldwide have extensively explored their involvement in various pathological conditions, including Alzheimer’s disease (AD), Parkinson’s disease (PD), multiple sclerosis (MS), and acute brain injury (Ding et al., 2022; Shevchuk et al., 2022; Wang H.-Q. et al., 2022; Wang et al., 2025). Existing studies indicate that microglia, by activating inflammasomes, trigger the excessive release of pro-inflammatory cytokines and pyroptosis, exacerbating neuroinflammation, causing neuronal damage, and potentially playing a key role in the onset and progression of numerous neurodegenerative diseases (Heneka et al., 2013; Wu et al., 2014; Lee J. W. et al., 2021; Yuan et al., 2021). Further research has revealed that inhibiting microglial pyroptosis and inflammasome activation plays a therapeutic role in modulating neuroimmune responses, promoting neural repair, and maintaining the blood-brain barrier (Wang M. et al., 2023; Cheng et al., 2024; Lu et al., 2024). Therapeutic strategies targeting microglial pyroptosis and inflammasomes, such as specific small-molecule inhibitors, antibody therapies, and traditional Chinese medicine, have shown promise in animal models, though significant challenges remain for their clinical translation (Jing et al., 2022; Xia et al., 2022; Xiong et al., 2022).

The research on microglial pyroptosis and inflammasomes has made significant progress, contributing to growing and dynamic articles. A systematic and comprehensive analysis of the articles in this field can reveal the current research status and hotspots, emerging trends, and future directions, which is of considerable importance for further studies and clinical applications. This study systematically analyzed relevant articles from the past decade on microglial pyroptosis and inflammasomes in the Web of Science Core Collection (WoSCC) database, aiming to promote in-depth research on microglial pyroptosis and inflammasomes in neurological diseases and to provide a theoretical foundation for the clinical treatment of these diseases.

## 2 Materials and methods

**FIGURE 1:**
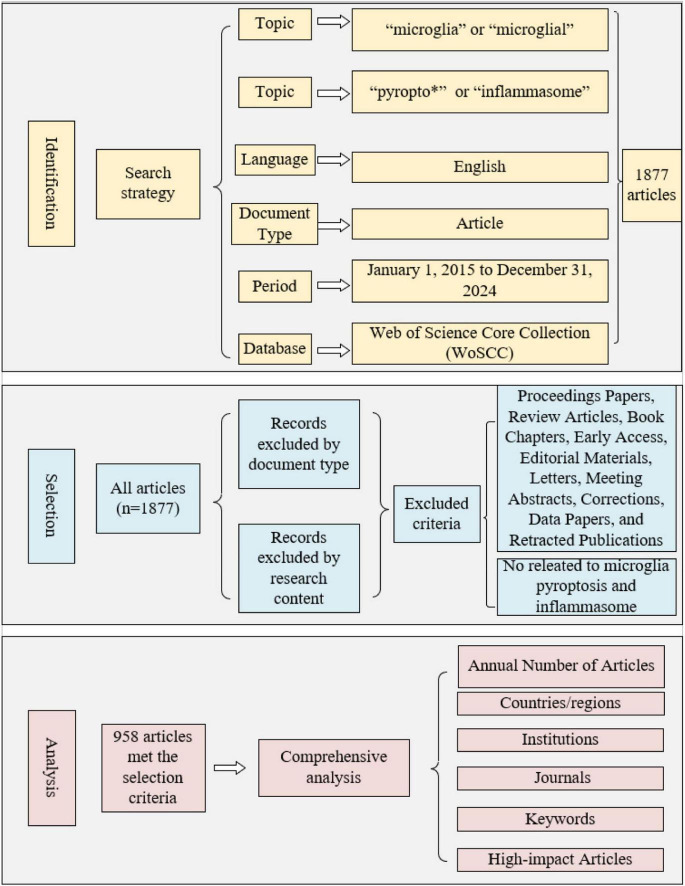
Frame flow diagram for the comprehensive analysis of 10-year articles.

This study utilized the WoSCC database as the data source to identify and analyze high-quality articles in the field of microglial pyroptosis and inflammasomes. The search strategy was based on reviews, meta-analyses, and relevant articles (Chen et al., 2020; Jia et al., 2023; Zhang Y. et al., 2024). The specific search formula used was: Topic = (“microglia” or “microglial”) AND (“pyropto*” or “inflammasome”). The search was limited to English-language articles published between January 1, 2015, and December 31, 2024. Based on this, the following exclusion criteria were established: (1) Document type: conference papers, reviews, book chapters, early open-access articles, editorial materials, letters, conference abstracts, corrections, data papers, and retracted articles; (2) Content relevance: articles not directly related to the research of microglial pyroptosis and inflammasomes. The screening process was conducted independently by two researchers, with final confirmation by BXM in case of any disputes. The selected articles were analyzed using statistical tools such as CiteSpace 6.3.1 and the WoSCC database, covering aspects such as annual article number, countries/regions, institutions, journals, research categories, and keywords. Furthermore, high-impact articles were subjected to a detailed analysis. The full procedure is shown in Figure 1.

## 3 Results

### 3.1 Annual article number analysis

**FIGURE 2:**
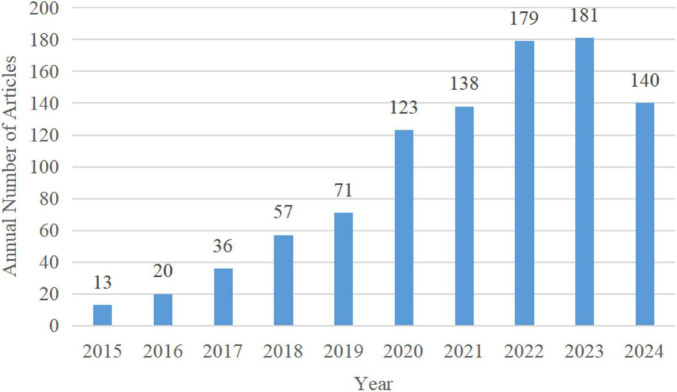
Annual article number in the field of microglial pyroptosis and inflammasomes.

After identifying and screening, a total of 958 articles were included in this study. Over the past decade, the number of articles published on microglial pyroptosis and inflammasomes has surged dramatically, with a notable increase in publications in 2019 and 2022. Since 2020, the annual article number has remained high, consistently exceeding 120 articles per year. The annual article number from 2015 to 2024 in the field of microglial pyroptosis and inflammasomes is shown in Figure 2. (Note: The 2024 data may appear slightly lower due to the inclusion delay of some articles in the database.)

### 3.2 Countries or regions analysis

**FIGURE 3:**
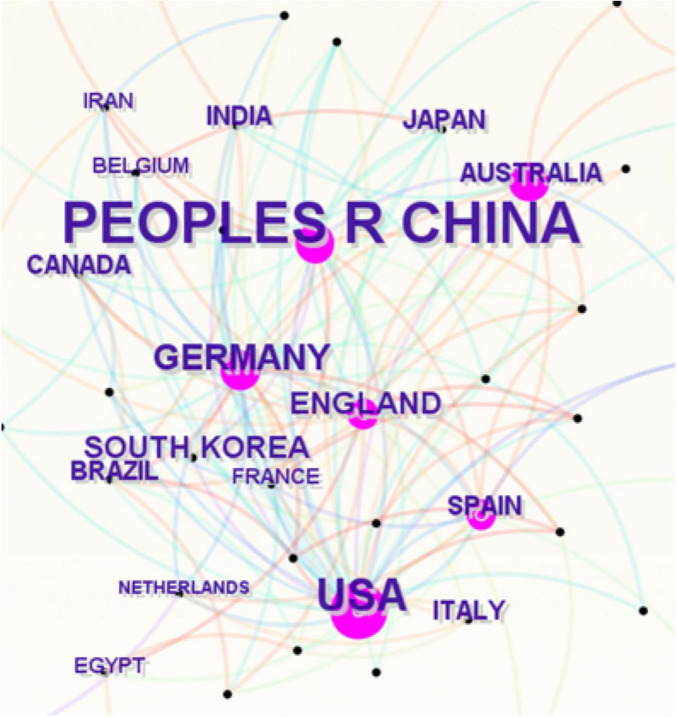
Collaboration network of countries or regions in the field of microglial pyroptosis and inflammasomes.

The included articles originated from 56 countries and regions, with China (664 articles), the United States (147 articles), and Germany (45 articles) leading in article number in this field. Figure 3 presents a collaboration network diagram based on CiteSpace’s default settings, where the size of each label and node is proportional to the number of articles, the connections between nodes represent collaborative relationships, and the density of the connections reflects the intensity of cooperation. In terms of article impact, the H-index is widely used as a standard for evaluating the influence and academic quality of articles, while centrality measures the status, closeness, and strength of cooperation of different countries or regions in the research collaboration network. China had the highest H-index (68) but a low centrality value of 0.02. The United States ranked second with an H-index of 48 and the highest centrality at 0.68. Specific details are shown in Table 1.

**TABLE 1:** The top 10 countries or regions with the highest article number on microglial pyroptosis and inflammasomes.

Rank	Countries or regions	Counts	Centrality	H-index
1	China	664	0.02	68
2	United States	147	0.68	48
3	Germany	45	0.01	26
4	England	32	0.02	14
5	South Korea	31	0.01	16
6	Australia	19	0.00	14
7	Italy	16	0.00	12
8	Brazil	15	0.00	9
9	Japan	15	0.00	10
10	Spain	15	0.00	11

### 3.3 Institutions analysis

**FIGURE 4:**
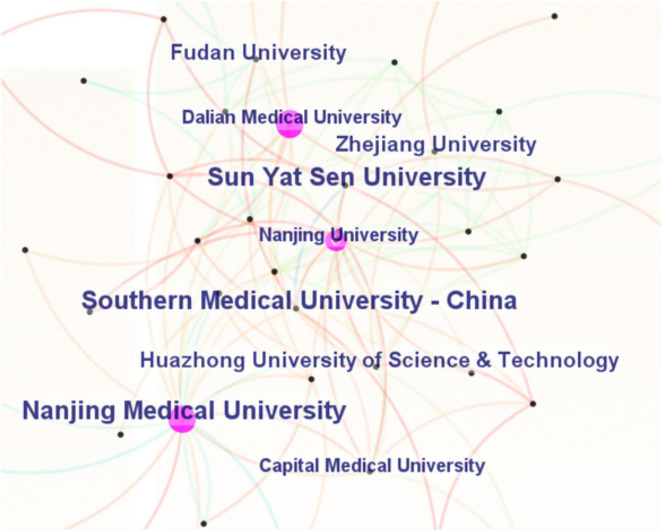
Collaboration network of institutions in the field of microglial pyroptosis and inflammasomes.

Table 2 presents the top 10 institutions in descending order of article volume. Southern Medical University (China) ranks first with 47 articles and an H-index of 19, followed by Nanjing Medical University (42 articles, H-index of 23) and Sun Yat-sen University (39 articles, H-index of 21). All of the top 10 institutions are located in China. Figure 4 shows the collaboration network among these institutions, where the size of each node and label represents the number of articles produced by the institution, and the connections between nodes represent collaborative relationships.

**TABLE 2:** The top 10 institutions with the highest article number on microglial pyroptosis and inflammasomes.

Rank	Institution	Country or regions	Counts	H-index
1	Southern Medical University	China	47	19
2	Nanjing Medical University	China	42	23
3	Sun Yat-sen University	China	39	21
4	Fudan University	China	31	16
5	Huazhong University of Science and Technology	China	31	16
6	Capital Medical University	China	23	9
7	Zhejiang University	China	23	12
8	Nanjing University	China	22	11
9	Shanghai Jiao Tong University	China	22	16
10	Shandong University	China	21	10

### 3.4 Journals and research categories analysis

Table 3 presents information on the top 10 journals in terms of article numbers in the field of microglial pyroptosis and inflammasomes. The *Journal of Neuroinflammation, International Immunopharmacology*, and *Molecular Neurobiology* published the most articles, with 59, 47, and 25 articles, respectively. The main research categories included neuroscience, immunology, pharmacology and pharmaceutics, as well as biochemistry and molecular biology.

**TABLE 3:** The top 10 journals with the highest article number on microglial pyroptosis and inflammasomes.

Rank	Journals	Research categories	Counts	Journal impact factor 2023
1	*Journal of Neuroinflammation*	Immunology; neurosciences	59	9.3
2	*International Immunopharmacology*	Immunology; pharmacology and pharmacy	47	4.8
3	*Molecular Neurobiology*	Neurosciences	25	4.6
4	*Frontiers in Immunology*	Immunology	22	5.7
5	*Brain Behavior and Immunity*	Immunology; neurosciences; psychiatry	16	8.8
6	*International Journal of Molecular Sciences*	Biochemistry and molecular biology;chemistry, multidisciplinary	14	4.9
7	*Brain Research*	Neurosciences	13	2.7
8	*Frontiers in Pharmacology*	Pharmacology and pharmacy	13	4.4
9	*CNS Neuroscience and Therapeutics*	Neurosciences; pharmacology and pharmacy	12	4.8
10	*Neurochemical Research*	Biochemistry and molecular biologyneurosciences	12	3.7

### 3.5 Keywords analysis

**FIGURE 5:**
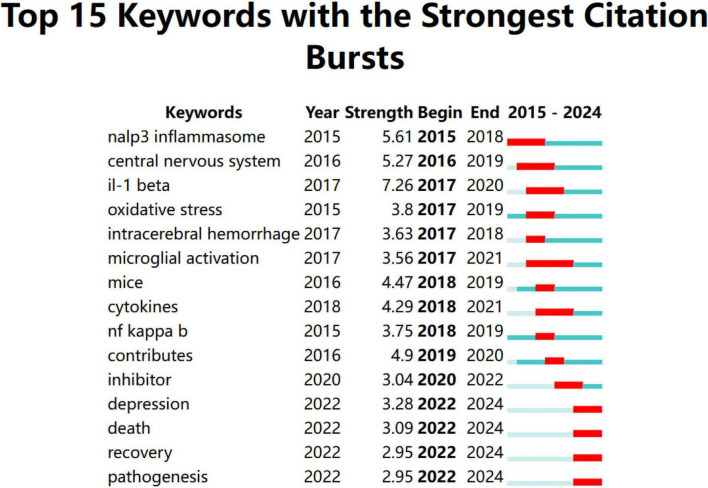
Keywords with the strongest citation bursts for articles on microglial pyroptosis and inflammasomes.

Keywords analysis was performed using CiteSpace software with the following parameters: “Years per slice” = 1, “Top N%” = 10.0%, and “Minimum duration” = 1. Figure 5 illustrates the temporal evolution of each keyword, with “strength” indicating the intensity of keyword bursts. The keyword “il-1 beta” exhibited the highest burst strength, at 7.26. The red squares in the figure represent the duration of keyword bursts, and keywords that persist until 2024 reflect the current research hotspots. “Microglial activation” is the keyword with the longest burst duration. Keywords that emerged between 2022 and 2024 included “depression,” “death,” “recovery,” and “pathogenesis.”

### 3.6 High-impact articles analysis

Citation count is often an indicator of an article’s recognition within the academic community. Highly cited articles tend to carry substantial academic influence, representing significant research achievements in their field. Table 4 lists the top 10 articles with the highest citation in the field of microglial pyroptosis and inflammasomes. These studies predominantly investigated the mechanisms underlying microglial pyroptosis and inflammasome activation (particularly NLRP3) in neurodegenerative diseases and central nervous system inflammation, as well as potential therapeutic interventions. Regarding mechanistic studies, these works have elucidated the activation of the NLRP3 inflammasome in microglial cells in diseases such as AD, PD, and MS. This activation triggers the release of inflammatory cytokines IL-1β and IL-18, exacerbating neuronal damage through pyroptosis mediated by GSDMD. On the therapeutic front, NLRP3 inhibitors (e.g., MCC950), quercetin, MicroRNA-7, and melatonin have been shown to inhibit NLRP3 activation or pyroptosis, thereby reducing neuroinflammation and improving cognitive and motor functions. These findings highlighted the pivotal role of microglial pyroptosis and inflammasome activation in neurodegenerative diseases, offering potential therapeutic targets for related conditions.

**TABLE 4:** Top 10 articles with the highest citation in the field of microglial pyroptosis and inflammasomes.

Rank	Title of article	DOI	Times cited	Interpretation of the findings
1	Inflammasome inhibition prevents α-synuclein pathology and dopaminergic neurodegeneration in mice	10.1126/scitranslmed.aah4066	540	The activation of the NLRP3 inflammasome in microglial cells drives neuroinflammation and dopaminergic neuronal damage in Parkinson’s disease. Inhibition of NLRP3 can alleviate disease progression and improve motor function.
2	Inhibiting the NLRP3 inflammasome with MCC950 promotes non-phlogistic clearance of amyloid-β and cognitive function in APP/PS1 mice	10.1016/j.bbi.2016.12.014	389	The NLRP3 inflammasome inhibitor MCC950 can suppress microglial pyroptosis-associated inflammatory responses, promote Aβ clearance, and improve cognitive function in AD models.
3	MicroRNA-7 targets Nod-like receptor protein 3 inflammasome to modulate neuroinflammation in the pathogenesis of Parkinson’s disease	10.1186/s13024-016-0094-3	367	MicroRNA-7 alleviates Parkinson’s disease-related neuroinflammation and dopaminergic neuronal damage by inhibiting microglial NLRP3 inflammasome activation, offering a potential novel therapeutic target.
4	Caspase-1 inhibition prevents glial inflammasome activation and pyroptosis in models of multiple sclerosis	10.1073/pnas.1722041115	358	In MS, microglia and oligodendrocytes promote inflammatory demyelination through GSDMD-mediated pyroptosis. Inhibiting pyroptosis can improve neuronal damage and alleviate disease progression.
5	Melatonin Attenuates LPS-Induced Acute Depressive-Like Behaviors and Microglial NLRP3 Inflammasome Activation Through the SIRT1/Nrf2 Pathway	10.3389/fimmu.2019.01511	342	Melatonin alleviates oxidative stress and pyroptosis, improves LPS-induced depressive-like behavior, and provides a novel therapeutic approach for depression by inhibiting the activation of the NLRP3 inflammasome in microglia.
6	NLRP3 Inflammasome Is Expressed and Functional in Mouse Brain Microglia but Not in Astrocytes	10.1371/journal.pone.0130624	285	Microglia in the brain can secrete IL-1β through the activation of the NLRP3 inflammasome, a capability not shared by astrocytes. The role of microglia is especially crucial under neuroinflammatory conditions.
7	NLR members NLRC4 and NLRP3 mediate sterile inflammasome activation in microglia and astrocytes	10.1084/jem.20150237	282	Lysophosphatidylcholine (LPC) triggers neuroinflammatory responses by activating the NLRP3 and NLRC4 inflammasomes in both microglial and astrocytic cells. This activation plays a crucial role in neurodegenerative diseases and demyelination, influencing the proliferation of these glial cells.
8	Aggregated Tau activates NLRP3-ASC inflammasome exacerbating exogenously seeded and non-exogenously seeded Tau pathology *in vivo*	10.1007/s00401-018-01957-y	280	Aggregated Tau is internalized by microglia and sorted via lysosomal pathways, activating the NLRP3-ASC inflammasome. This process exacerbates both exogenous and endogenous propagation of Tau pathology, offering a new potential therapeutic target for AD.
9	MPTP-driven NLRP3 inflammasome activation in microglia plays a central role in dopaminergic neurodegeneration	10.1038/s41418-018-0124-5	271	Activation of the NLRP3 inflammasome in microglia is critical for dopaminergic neuronal loss and the subsequent motor deficits observed in the MPTP-induced PD mouse model.
10	Quercetin hinders microglial activation to alleviate neurotoxicity via the interplay between NLRP3 inflammasome and mitophagy	10.1016/j.redox.2021.102010	255	Quercetin protects neurons from LPS-induced toxic damage by promoting mitochondrial autophagy, reducing mitochondrial reactive oxygen species (mtROS) accumulation, and inhibiting the activation of NLRP3 inflammasomes in microglia. This suggests a potential therapeutic strategy for diseases associated with neuroinflammation.

## 4 Discussion

### 4.1 General data

Over the past decade, there has been a significant increase in the annual article number in the field of microglial pyroptosis and inflammasomes, particularly since 2020, with the number consistently remaining high. This trend indicates a growing interest and attention toward this area of research. In terms of national and regional contributions, China, the United States, and Germany led in article number, with both China and the United States demonstrating substantial academic influence. Although China ranked first in both article number and influence, it lacked the depth and breadth of international collaborations. Among institutions, Southern Medical University (China), Nanjing Medical University, and Sun Yat-sen University have shown outstanding performance in terms of article number and academic impact, holding leading positions. Regarding journal distribution, the *Journal of Neuroinflammation*, *International Immunopharmacology*, and *Molecular Neurobiology* were the journals with the highest article numbers, highlighting their significant roles within the field. High-impact articles mainly focused on investigating the pathological mechanisms of microglial pyroptosis and inflammasome activation in neurodegenerative diseases and central nervous system inflammation, as well as their potential therapeutic interventions. Researchers and clinicians should pay particular attention to countries, regions, institutions, and journals with high article frequency and academic influence, especially to emerging results from these sources. This will facilitate further scientific progress in the field of microglial pyroptosis and inflammasomes and provide essential theoretical support and practical foundations for exploring the pathological mechanisms of neurological diseases and their clinical interventions.

### 4.2 Research hotspots

An analysis of emerging keywords reveals the evolving research hotspots in the field of microglial pyroptosis and inflammasomes, as reflected in the top 15 keywords with the strongest citation bursts. The emerging keyword “nalp3 inflammasome” (also known as NLRP3 inflammasome) indicates an increasing focus on the mechanisms of microglial inflammasomes. Lai et al. (2018) found that the NALP3 inflammasome in microglia negatively regulated autophagy through Caspase-1-mediated cleavage of TRIF, playing a critical role in prion disease-related neuroinflammation. The appearance of the keyword “central nervous system” signifies that microglial pyroptosis and inflammasome activation predominantly affected the CNS (Cao et al., 2024; Chen et al., 2024; Wang et al., 2024). The keyword “cerebral hamorrhage” highlights studies on how inhibiting microglial pyroptosis and inflammasome activation could alleviate inflammation following cerebral hemorrhage (Bao et al., 2023; Lei et al., 2023; Liu et al., 2023). Keywords such as “interleukin-1β,” “oxidative stress,” “microglial activation,” “cytokines,” and “NF-kappa B” are all critical factors, inflammatory mediators, and key pathway proteins involved in the process of microglial pyroptosis and inflammasome activation, reflecting a growing depth and clarity in mechanistic research within this field (Bai et al., 2018; Jin et al., 2019; Li et al., 2021). The keyword “inhibitor” refers to inhibitors of inflammatory mediators or pathway proteins, indicating an increasing emphasis on exploring interventions and therapies (Henry et al., 2020; Chen et al., 2021; Yan et al., 2023).

Keywords emerging in 2022 and continuing through 2024 include “depression,” “death,” “recovery,” and “pathogenesis,” reflecting the latest research hotspots.

“Depression” is a priority disease in microglia pyrolysis and inflammasome research, and understanding its pathological mechanisms is crucial for the diagnosis and treatment of depression. In recent years, research into the molecular mechanisms underlying microglial pyroptosis and inflammasomes has advanced, particularly regarding their role in triggering neuroinflammation and neuronal damage in depression. Liu et al. (2024) have shown that AdipoRon, by promoting mitochondrial autophagy, inhibited NLRP3 inflammasome activation in microglia, protecting hippocampal neurons from microglial toxicity and exerting antidepressant effects. Additionally, inhibiting microglial pyroptosis has been identified as a promising therapeutic target for improving depression. Gao Z. et al. (2024) demonstrated that microglial pyroptosis and NLRP3 inflammasome activation played central roles in chronic stress-induced hippocampal damage and depressive-like behaviors. Furthermore, they revealed that melatonin can significantly alleviate chronic stress-induced pyroptosis by inhibiting the Cathepsin B/NLRP3 signaling pathway, thereby ameliorating depressive symptoms. Moreover, low-dose esketamine has been shown to regulate the GSK-3β/NLRP3 signaling pathway, exhibiting neuroprotective effects in LPS-induced depressive mice (Strawbridge et al., 2017). These studies all highlight the significant role of microglial pyroptosis and inflammasomes in the pathological mechanisms and treatment of depression.

“Death” and “recovery” refer to neuronal death and functional recovery, which are central in studies of microglial pyroptosis and inflammasome activation, serving as critical entry points for understanding the pathological processes underlying CNS diseases. Current research is primarily focused on pharmacological interventions targeting pyroptotic pathways to attenuate cell death and promote functional recovery. Inhibiting the activation of the NLRP3 inflammasome or caspase-1 activity in microglia has been shown to reduce pyroptosis, alleviate associated neuroinflammation, decrease neuronal cell death, and enhance neurofunctional recovery (Cao et al., 2024; Zhang B. et al., 2024). Specific compounds, such as VX-765, have shown significant neuroprotective effects by regulating reactive oxygen species (ROS) levels and mitochondrial function, thereby preventing the cell death process associated with microglial pyroptosis (Sun et al., 2020). Moreover, dichloroacetate (DCA) has been reported to reduce neuronal death induced by sepsis-associated encephalopathy (SAE) and to improve cognitive function during the recovery phase in LPS-treated mice (Huang et al., 2024). In recent years, increasing attention has been directed toward the mechanisms, clinical applicability, and long-term effects of these interventions, particularly regarding functional recovery following reduced cell death. These findings provide critical insights into the dynamic mechanisms and regulation of neuronal death and recovery associated with microglial pyroptosis and inflammasome and offer a foundation for novel therapeutic strategies (Gu et al., 2022).

Research on the “pathogenesis” has elucidated the specific roles of microglial pyroptosis and inflammasomes in neuroinflammation and neurological diseases. In neuropathic pain, microglia undergo pyroptosis through the activation of the NLRP3 inflammasome, which triggers the release of pro-inflammatory cytokines, thereby exacerbating hyperalgesia (Gao X. et al., 2024). In cerebral small vessel disease (CSVD), NLRP3 inflammasome activation in microglia is closely associated with blood-brain barrier disruption, white matter injury, and cognitive decline, with the inflammatory pathway it mediates potentially playing a central role in the pathogenesis of CSVD (Zhang M. et al., 2024). Microglial pyroptosis and inflammasome activation are core drivers of inflammation amplification and pathological deterioration. Furthermore, in-depth research into pathogenic mechanisms provides directions for targeted therapies. The investigation of small molecule compounds and their regulatory effects on pyroptosis and inflammasome activation has become a prominent research focus for targeted therapeutic intervention in various diseases. For instance, TBK1 has been shown to alleviate diabetic neuropathy by inhibiting microglial pyroptosis (Liao et al., 2024). DL-3-n-butylphthalide (NBP) exerts negative regulation on pyroptosis in stroke, while formoterol mitigates pyroptosis through autophagic pathways (Ge et al., 2024). The pathogenesis of microglial pyroptosis and inflammasome activation has become a frontier and a hot topic in exploring pathological mechanisms and intervention strategies for neurological diseases.

### 4.3 High-impact research findings

The findings of high-impact articles represent significant advancements in the field, with profound implications for current and future research. These articles primarily focused on the mechanisms of microglial pyroptosis and inflammasome activation, elucidating their pathological roles in neurodegenerative diseases such as AD, PD, and MS, and exploring potential therapeutic intervention strategies.

#### 4.3.1 Mechanisms of pyroptosis and inflammasome activation in microglia

Microglia are resident cells in the central nervous system (CNS) that perform a variety of functions, including brain development, maintenance of neural homeostasis, immune surveillance, cytokine secretion, injury repair, and synaptic pruning and remodeling (Marschallinger et al., 2020; Escoubas and Molofsky, 2024). Under resting conditions, microglia dynamically monitor the local immune environment to maintain tissue homeostasis. When exposed to external stimuli, microglia rapidly activate and undergo phenotypic changes, differentiating into M1 pro-inflammatory (classically activated) and M2 anti-inflammatory and reparative (alternatively activated) phenotypes, playing a crucial regulatory role in the inflammatory response. In activated microglia, the inflammasome detects both endogenous and exogenous stress signals, leading to its upregulation and activation, which in turn triggers pyroptosis, exacerbating the inflammatory response and influencing the pathological processes of neurodegenerative diseases (Moonen et al., 2023; Li X. et al., 2024).

Inflammasomes can be classified into NLR family inflammasomes (e.g., NLRP3, NLRC4), AIM2 family inflammasomes (e.g., AIM2), and atypical inflammasomes (e.g., Pyrin). Different inflammasome receptors recognize distinct activation signals and trigger the downstream activation of caspase-1, which mediates the maturation and release of IL-1β and IL-18. This also leads to the cleavage of GSDMD, resulting in the release of N-terminal GSDMD (GSDMD-N), which forms membrane pores and induces the release of cellular contents, thereby initiating pyroptosis (Wang et al., 2021; Fu et al., 2024). Under the activation of caspase-1 and caspase-11, the aspartate residue of GSDMD is cleaved, releasing the N-terminal fragment of GSDMD-N, which forms membrane pores and induces pyroptosis. Additionally, other members of the GSDM family, such as GSDMA and GSDME, also play a role in inducing pyroptosis (Wang et al., 2020; Vasudevan et al., 2023).

In neurodegenerative diseases and CNS inflammation-related disorders, the NLRP3 inflammasome and its mediated activation of GSDMD play significant roles and have become a major focus of research. The NLRP3 inflammasome is a multi-protein complex consisting of NLRP3, apoptosis-associated speck-like protein (ASC), and caspase-1. Under stimuli such as ATP release, potassium ion efflux, reactive oxygen species (ROS) generation, and lysosomal rupture, NLRP3 is activated and recruits caspase-1, which then cleaves and activates pro-IL-1β and pro-IL-18. In addition, caspase-1 induces the cleavage of GSDMD, and the GSDMD-N inserts into the cell membrane, forming pores that release mature IL-1β and IL-18 while disrupting membrane integrity, thus triggering pyroptosis and amplifying the local inflammatory response (Coll et al., 2022; Kinra et al., 2022). This mechanism significantly accelerates the pathological spread and tissue damage in various diseases, including AD, Parkinson’s disease, and brain injuries, thereby greatly impacting microglia-mediated neuroinflammation and disease progression (Lee J. H. et al., 2021; Wang L. et al., 2022).

#### 4.3.2 Pathological functions of microglial pyroptosis and inflammasome activation in neurodegenerative diseases

As aging progresses, human microglia exhibit significant alterations in both morphology and function. The gene expression of aging microglia is altered in several aspects, such as actin dynamics, cell adhesion, DNA damage, and phagocytic function. Morphological changes include thinner, fragmented processes and ferritin accumulation. In particular, aging enhances the inflammatory phenotype of microglia. Activated subpopulations upregulate pro-inflammatory cytokines, including Ccl3, Ccl4, Tnf, and Il1b, promoting persistent neuroinflammation. Specific microglial clusters, such as OA2 and ARM subpopulations, show increased expression of inflammation-related genes with age (Mecca et al., 2018; Antignano et al., 2023). This suggests excessive immune activation, which is linked to age-related neurodegenerative diseases. In high-impact articles, PD, AD, and MS are the primary focus of research. In these diseases, aging microglia are excessively activated and polarized to the M1 phenotype (Colonna and Butovsky, 2017; Yu et al., 2023). This enhances inflammasome activation, and cytokine release, and induces pyroptosis, which amplifies inflammation and worsens neuronal damage, driving disease progression.

PD is a common neurodegenerative disorder primarily characterized by motor dysfunction, including bradykinesia, tremors, and issues related to gait and balance (Sveinbjornsdottir, 2016). The pathological hallmark of Parkinson’s disease primarily involves the loss of dopaminergic neurons in the substantia nigra pars compacta (SNpc), resulting in dopamine deficiency in the striatum, along with the formation of intracellular inclusions containing α-synuclein aggregates (Poewe et al., 2017). Recent studies have shown that microglial activation and neuroinflammatory responses are also crucial pathological features of Parkinson’s disease (Zhang et al., 2017; Badanjak et al., 2021; Forloni, 2023). aggregated α-synuclein (α-syn) acts as a Damage-Associated Molecular Pattern (DAMP) that directly activates microglial immune receptors, such as Toll-like receptors (TLRs), facilitating the expression of NF-κB and other inflammatory pathways, as well as inflammasome activation. Additionally, α-synuclein can be internalized by microglial cells, damaging mitochondrial function, and leading to the generation of mitochondrial DNA (mtDNA) and mitochondrial-derived reactive oxygen species (mtROS), which further activate inflammasomes. Both of these mechanisms contribute to the activation of inflammasomes like NLRP3 in microglial cells, inducing the production and release of IL-18, IL-1β, and active caspase-1, which may even trigger pyroptosis, further aggravating neuroinflammation and damaging dopaminergic neurons. Furthermore, inflammatory factors secreted by microglial cells, such as IL-1β, IL-6, IFN-γ, ROS, NO, and COX2, promote the aggregation of α-synuclein, creating a positive feedback loop that intensifies neuronal damage and ultimately leads to motor dysfunction. Key signaling pathways involved in inflammasome activation in microglial cells during Parkinson’s disease progression include TLRs/NF-κB/NLRP3, TLR/NLRP3/Caspase-1, and NF-κB/AP-1/Nrf2 (Zhou et al., 2016; Gordon et al., 2018; Lee et al., 2019). MCC950 and miRNA-7 inhibitors, by suppressing the activation of the NLRP3 inflammasome within microglial cells, can alleviate neuroinflammation and neuronal damage associated with Parkinson’s disease, offering new potential therapeutic targets for the treatment of PD.

AD is an age-related neurodegenerative disorder characterized by progressive cognitive impairment, memory loss, and abnormal behaviors (Farlow, 1998). Its neuropathological hallmarks include amyloid β (Aβ) plaques and neurofibrillary tangles (NFTs) formed by hyperphosphorylated tau protein aggregates, accompanied by neuroinflammation and loss of neurons and synapses (Guo et al., 2020). Neuroinflammation is involved throughout the progression of AD, with persistent microglia-mediated neuroinflammation being a major contributor to the neurodegenerative process and cognitive deficits in AD patients (Kinney et al., 2018; Leng and Edison, 2021; AmeliMojarad and AmeliMojarad, 2024). In the early stages of AD, activation of microglia helps in the clearance and phagocytosis of Aβ. However, as the disease progresses, the prolonged activation of microglia gradually impairs their normal phagocytic function and triggers a chronic inflammatory response. This response releases inflammatory mediators such as cytokines, complement components, chemokines, and free radicals, which exacerbate the pathological accumulation of Aβ and tau, further driving the progression of the pathology (Sebastian Monasor et al., 2020; Wang C. et al., 2023). Aβ activates microglia by binding to receptors on the microglial cell surface, such as CD14, TLR2, and TLR4, triggering the activation of several inflammatory pathways including NF-κB, JAK-STAT, and the NLRP3 inflammasome. This activation leads to the release of numerous inflammatory mediators, including cytokines, chemokines, and free radicals, which further promote Aβ accumulation, exacerbate neuroinflammation, and result in neuronal damage. Additionally, activation of the NLRP3 inflammasome further mediates pyroptosis, releasing ASC (apoptosis-associated speck-like protein), which forms ASC-Aβ fusion proteins. These not only facilitate the propagation of Aβ but also integrate into the NLRP3 inflammasomes of neighboring microglia, amplifying the inflammatory response and pyroptosis, thereby exacerbating neuronal damage (Venegas et al., 2017; Moonen et al., 2023; Hu et al., 2024). In AD, phosphorylated tau can also activate the NLRP3 inflammasome within microglia through receptors such as TLR4, further promoting the accumulation and spread of tau. The activation of the inflammasome leads to the release of pro-inflammatory cytokines from microglia, which increases tau phosphorylation, thereby exacerbating the formation of neurofibrillary tangles (NFTs). Additionally, this activation enhances the seeding effect of tau, facilitating its propagation between neurons (Merighi et al., 2022; Ayyubova, 2023). In this study, two high-impact articles demonstrate that the activation of the NLRP3 inflammasome exacerbates Aβ accumulation and neurofunctional damage in AD mouse models. Additionally, aggregated tau proteins promote the progression of tau pathology by activating the NLRP3-ASC inflammasome in microglia. And MCC950 may serve as a potential new intervention strategy for AD (Dempsey et al., 2017; Stancu et al., 2019).

MS is an immune-mediated chronic inflammatory demyelinating disease that primarily affects young individuals and is a common non-traumatic disabling neurological disorder. Its clinical manifestations typically include abnormalities in motor and sensory functions as well as cognitive impairment. Common symptoms include optic neuritis, brainstem, and spinal cord syndromes. The pathological features of MS are characterized by immune-mediated inflammatory responses, demyelination, and axonal damage, which contribute to the neurodegenerative processes (Katz Sand, 2015; Oh et al., 2018). In the early stages of the disease, axons are typically relatively preserved. However, as the disease progresses, widespread activation of microglia and irreversible demyelination and axonal damage gradually worsen, ultimately leading to the progressive loss of neurological function and the accumulation of disability (Compston and Coles, 2008; Hagemeier et al., 2012). Microglia promote neuroinflammation, myelin damage, and the pathological progression of MS through the activation of the NLRP3 inflammasome and pyroptosis. In response to intracellular and extracellular stimuli, microglia activate inflammasomes such as NLRP3, releasing pro-inflammatory cytokines like IL-1β and IL-18. These cytokines not only exacerbate local inflammatory responses but also regulate the phenotypic transformation of immune cells, driving the pathological progression of MS (Zhang et al., 2018; Fan et al., 2024). Studies have shown that the activation of the NLRP3 inflammasome is closely associated with the severity of myelin damage and neuroinflammation in MS patients and experimental autoimmune encephalomyelitis (EAE) mouse models. The application of NLRP3 small molecule inhibitors, such as MCC950, effectively suppresses inflammasome activation in microglia and macrophages. Additionally, it mitigates axonal damage in demyelinating mouse models (Dempsey et al., 2017; Soraci et al., 2023). Microglial pyroptosis is mediated by the activation of caspases 1, 3, and 7, as well as the cleavage of GSDMD, leading to cell membrane rupture and the release of pro-inflammatory factors. This process further exacerbates local neuroinflammation, promotes myelin destruction, and disrupts neuronal signaling (McKenzie et al., 2020; Pollock et al., 2024). High-impact articles indicate that the Caspase-1 inhibitor VX-765 significantly alleviates inflammasome activation and pyroptosis in the EAE model, reducing axonal damage and improving neurological function (McKenzie et al., 2018). Microglial pyroptosis and inflammasomes such as NLRP3 play a crucial role in the progression of MS. Targeting these pathways by inhibiting the activation of the NLRP3 inflammasome or pyroptosis may represent a novel therapeutic strategy for MS in the future (Galloway et al., 2022; Naeem et al., 2022).

### 4.4 Future research trends

Over the past decade, articles on microglial pyroptosis and inflammasomes have increased significantly, reflecting growing academic interest in this field. Keywords such as “depression,” “death,” “recovery,” and “pathogenesis” and high-impact articles not only highlight the current research hotspots but also point toward future research trends. Research on microglial pyroptosis and inflammasomes will focus on pivotal areas, including mechanistic exploration, dynamic regulation, targeted intervention, and clinical translation. Firstly, mechanistic studies in this field will further investigate the activation of inflammasomes and pyroptosis in microglia, aiming to elucidate their regulatory characteristics in various pathological environments (Liu et al., 2024). The research will also explore how these processes contribute to different diseases (Zhang M. et al., 2024), particularly their specific roles in neurodegenerative diseases and their interactions with other cells. Notably, the phenotypic and functional changes of aging microglia, as well as their specificity in inflammatory responses, will become a crucial focus of future research. Secondly, in terms of therapeutic strategies, targeted interventions for microglial pyroptosis and inflammasomes will increasingly focus on the optimization of existing inhibitors and the development of novel molecular targets. Additionally, exploring combination therapies that regulate pyroptosis alongside other pathological pathways will be a significant area of focus (He et al., 2023; Liao et al., 2024). Finally, the clinical application and efficacy of small molecule targeted therapeutics, including pyroptosis and inflammasome inhibitors, in diseases such as PD, AD, and MS, will emerge as a critical area of research, aiming to advance the treatment of neurological disorders (Gao X. et al., 2024).

### 4.5 Limitations

This study has several limitations. Firstly, to ensure high-quality articles inclusion, and based on previous studies (Wang R. et al., 2022; Zhao et al., 2022; Feng et al., 2023), the database was restricted to the WoSCC database, which may have led to an incomplete collection of relevant articles. Secondly, the search was limited to English-language articles, which could have excluded valuable research published in other languages. Thirdly, only articles were included, potentially overlooking important contributions from other types. Fourthly, publication time was restricted to capture the latest hotspots, which may have resulted in incomplete coverage of relevant articles. Additionally, there are several limitations in the current research on microglial pyroptosis and inflammasomes: (1) In terms of mechanistic research, while the significant role of microglial pyroptosis and inflammasome activation in neurological diseases has been demonstrated, the interactions between microglia and other cells remain unclear. In particular, the expression and functional changes of aging microglia in neurodegenerative diseases and their specificity in inflammatory responses have not been fully clarified, and there is a lack of research on human aging microglia. Additionally, the interplay between pyroptosis and other forms of cell death, such as autophagy and apoptosis, as well as their associations with other pathological processes, require further in-depth exploration. (2) Existing animal models and *in vitro* cell lines serve as a foundation for research; however, they cannot fully replicate the complexity of human diseases, and interspecies variability may affect the generalizability of the experimental results. (3) Targeted interventions remain challenging. Existing inhibitors lack sufficient specificity and may adversely affect the immune system. (4) Diagnostic and detection methods for pyroptosis and inflammasome are not well-established, with a lack of highly sensitive and specific biomarkers to accurately assess the occurrence and extent of pyroptosis and inflammasome activation, complicating clinical assessment and diagnosis. (5) Clinical translation is lagging. Despite progress in basic research, the clinical translation of these findings is still in its early stages, and many potential therapeutic strategies have not been thoroughly validated, resulting in a slow translation into effective treatments.

## 5 Conclusion

This study provided a comprehensive analysis of the articles in the field of microglial pyroptosis and inflammasomes, revealing the current status, emerging hotspots, and future trends in the field, offering valuable guidance for researchers. Research on microglial pyroptosis and inflammasomes has been steadily growing, with China and the United States leading in this field. Southern Medical University (China) is recognized as the most influential institution. *Journal of Neuroinflammation*, *International Immunopharmacology*, and *Molecular Neurobiology* are leading journals. High-impact articles predominantly focus on the roles of microglial pyroptosis and inflammasomes in neurodegenerative diseases, such as AD, PD, and MS, as well as therapeutic interventions. Keywords like “depression,” “death,” “recovery,” and “pathogenesis” have emerged as research hotspots in this field. Microglial pyroptosis, through the activation of the NLRP3 inflammasome and the subsequent release of pro-inflammatory cytokines, contributes to neuroinflammation, neuronal death, and functional impairment, playing a crucial role in neurodegenerative diseases. However, specific research on aging microglia in these diseases is still insufficient. In recent years, targeted interventions, including MCC950, quercetin, MicroRNA-7, and melatonin, have shown therapeutic efficacy in modulating pyroptosis and inflammasome activity. Overall, research on microglial pyroptosis and inflammasomes has significantly deepened the understanding of neuroinflammation and the mechanisms underlying neurodegenerative diseases. Additionally, it has opened new avenues for precision treatment. Future studies will focus on further elucidating the mechanisms by which microglial pyroptosis and inflammasome activation contribute to various diseases and microenvironments, and their interactions with other cell types. Future research will build upon this foundation to further explore mechanisms and therapeutic strategies. The phenotypic and functional changes of aging human microglia, particularly their specific roles in inflammatory responses, will be a central focus in future research on neurodegenerative diseases. The in-depth exploration of these mechanisms will provide critical theoretical support for precision therapies and is expected to facilitate the development of more effective intervention strategies, ultimately improving disease progression.

## Data Availability

The original contributions presented in this study are included in this article/supplementary material, further inquiries can be directed to the corresponding authors.
